# Topographic Findings of the Porcine Cornea

**Published:** 2016

**Authors:** Jens HEICHEL, Frank WILHELM, Kathleen S. KUNERT, Thomas HAMMER

**Affiliations:** 1 Department of Ophthalmology, University Hospital of Martin Luther, University Halle-Wittenberg, Halle (Saale), Germany; 2 Augen im Zentrum, Greifswald, Germany; 3 Department of Ophthalmology, Helios Klinikum Erfurt, Erfurt, Germany; 4 Augenzentrum Frohe Zukunft, Halle (Saale), Germany

**Keywords:** Topography, Porcine, Cornea, Orbscan, Corneal Thickness, Refractive Power

## Abstract

The porcine eye is often used as an ex vivo animal model in ophthalmological research. It is well suited for investigations concerning refractive surgery; however, corneal topography data are scarce. This study investigated the corneal topography and pachymetry of the porcine eye to provide further reproducible data. We evaluated freshly enucleated porcine eyes (n = 16) by performing computerized corneal topographies (Orbscan® IIz, Bausch and Lomb, Rochester, NY, USA). We assessed the steepest and flattest keratometric powers (K1 and K2, units in diopters (D)), astigmatism (D), white-to-white (WTW) diameter (mm), thinnest point pachymetry (µm), anterior and posterior best-fit sphere (BFS) (D), refractive power of the anterior and posterior curvatures, and total refractive power of the cornea (D). The mean keratometric powers were 39.6 ± 0.89 D (K1) and 38.5 ± 0.92 D (K2), and the mean astigmatism was 1.1 ± 0.78 D. The mean WTW diameter was 13.81 ± 0.83 mm, and the mean corneal thickness was 832.6 ± 40.18 µm. The BFSs were 38.14 ± 0.73 D (anterior) and 42.56 ± 1.15 D (posterior), and the mean refractive powers were 43.27 ± 1.08 D (anterior) and -5.15 ± 0.20 D (posterior); therefore, the mean of the total refractive power was 38.16 ± 1.00 D. The topography and pachymetry of the porcine cornea showed a specific configuration differing from the human cornea. When using animal ex vivo models such as porcine corneas for experimental corneal surgery, findings such as these should be considered.

## INTRODUCION

Experimental surgery is important for the development of new surgical techniques. Therefore, ex vivo models that can simulate the human eye have been of great interest. The ability to train for ocular surgery in a nonstressful setting is an important component in the education of ophthalmologic surgeons (1). Today, different surgical models are available, ranging from computer simulation to animal models to human donor eyes (2-6). The porcine eye has been used for different experimental ophthalmologic studies, including cataract and corneal surgeries, glaucoma research, and even neuroretinal studies (5-8). As porcine and human ocular anatomy are quite similar, the porcine animal model is ideal for those demands. Corneal surgery studies have primarily been performed using porcine eyes (9). In that regard, the effect of corneal crosslinking and the impact of laser and its tissue modeling effect were revealed (10-16). Additional research has also measured aberrometry data of porcine eyes (17, 18). The aim of this paper was to provide detailed and reproducible data concerning the corneal topography of the porcine eye.

## MATEIALS AND METHODS

**Experimental Setting**

We used porcine eyes (Sus scrofa domesticus) of animals 7 to 8 months of age that were obtained from a local slaughterhouse. The eyes were not brewed. Examinations were performed within 12 hours after enucleation to avoid auto-digestive changes of the tissue. During that time, the specimens were stored in a wet chamber at 4°C. From 50 freshly enucleated pig eyes, we selected 16 that had been determined to have impeccable quality after a macroscopic evaluation. We excluded specimens with signs of trauma (e.g., corneal or scleral scars or corneal erosions) or malformation. Prior to topographic examination, the eyes were stored at room temperature 22 °C for 2 hours to reduce any possible swelling of the corneal stroma. The experimental protocol followed an established sequence approved in prior studies (12, 13).

For corneal mapping, the samples were fixed in a Spitznas vacuum bulb holder. We measured the intraocular pressure (IOP) using Schiotz impression tonometry. The IOP was set at 17.3 mmHg (5.0 scale units according to a 5.5-g load) for all specimens by varying the power of the vacuum of the bulb holder. We used the Orbscan® IIz (Bausch & Lomb, Rochester, NY, USA) to analyze the porcine corneal topographies. This optical mapping system utilizes an illuminated ring pattern (placid disc system) and a slit-shaped beam to detect corneal architecture. For each examination, we used the Zyoptix® mode, which averaged the three analyses. We detected the anterior and posterior elevation maps, surface power, and corneal thickness. 

**Collected Data**

The Zyoptix® mode of the Orbscan® IIz was used to detect the following parameters:

K1 and K2 (steepest and flattest keratometric power, in diopters (D)); Astigmatism (D); White-to-white (WTW) diameter (mm); Pachymetry at the thinnest point (µm);

Anterior best-fit sphere in D (anterior BFS, using a floating alignment; 9-mm fit zone); Posterior best-fit sphere in D (posterior BFS, using a floating alignment; 9-mm fit zone); Refractive power of the anterior cornea (D) (simulated using a paraxial eye model); Refractive power of the posterior cornea (D) (simulated using a paraxial eye model); Total refractive power of the cornea (D) (simulated using a paraxial eye model).

**Statistical Analysis**

Statistical analysis was performed using SPSS software (Version 21.0 for Windows; IBM Corporation, Armonk, NY, USA) and Excel software (Microsoft Corporation, Redmond, WA, USA). For each specimen, the results were averaged and the standard deviation was calculated. We generated boxplots to compare the different refractive power measurements. 

**Ethical Guidelines**

No donor tissue was used. The porcine eyes were obtained from a local slaughterhouse. Animals were not sacrificed for this study. All procedures were conducted in accordance with the ethical standards of the Helsinki Declaration of 1964, as revised in 2013. 

## RESULTS

**Figure 1:**
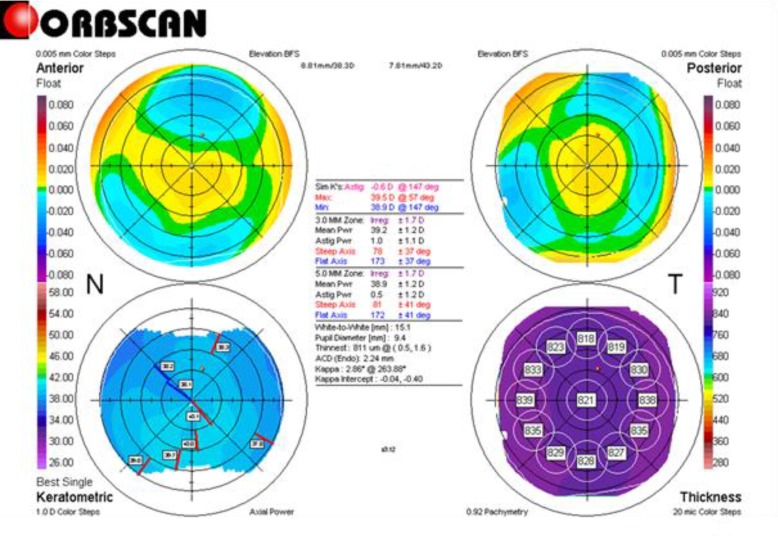
An Orbscan® IIz of a Porcine Cornea

**Figure 2:**
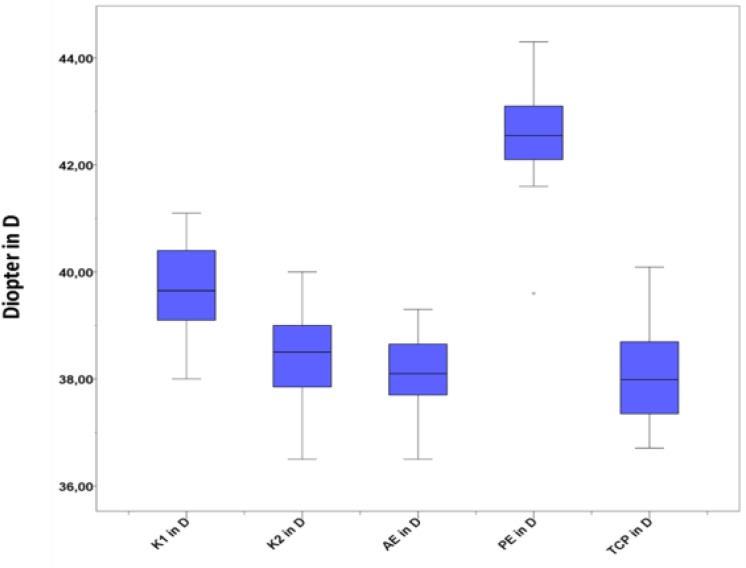
Boxplot of Keratometric Data

Abbreviations: K1 – highest keratometric power; K2 – lowest keratometric power; D – diopter; AE – anterior elevation best fit sphere; PE – posterior elevation best fit sphere; TCP – total corneal power.

Through this study, we examined corneal topographic characteristics of 16 porcine eyes. All specimens were measured using the Orbscan® IIz. The keratometric power of the steepest meridian ranged from 38.0 to 41.1 D (mean, 39.6); the flattest meridian ranged from 36.5 to 40.0 D (mean, 38.5). The astigmatic power varied from 0.3 to 3.4 D (mean, 1.1). The mean pachymetry measurement was 832.6 µm (range, 762 to 898). The WTW diameter had a mean of 13.8 mm (range, 12.3 to 15.1). All data are summarized in Table 1. Figure 1 shows an exemplary result screen of an Orbscan® IIz measurement performed in a porcine eye. Figure 2 depicts a boxplot of the main keratometric data.

**Figure 3:**
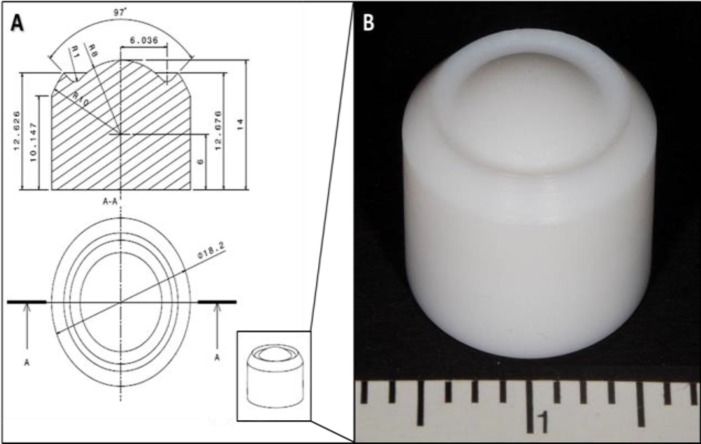
Calotte for Stabilizing Corneal Tissue

A calotte was constructed according to the topographic findings of the porcine cornea Figure 3. It consists of polytetrafluorethylene, a material that is very resistant to chemicals. We used this device for preparation of the corneal tissue for scanning electron microscopy.

Showing the anterior (top left) and posterior (top right) corneal powers; simulated keratometry (below left) and pachymetry (below right).

**Table 1:** Summary of the Orbscan® IIz Data for Porcine Eyes (n = 16)

**N°**	**K1 in D**	**K2 in D**	**Ast. In D**	**WTW in mm**	**Pachymetry in μm (thinnest)**	**Ant. Elev. BFS in D**	**Post. Elev. BFS in D**	**Ant. Cornea in D**	**Post. Cornea in D**	**TCP in D**
**1**	40.4	39.9	0.6	14.5	873	39.3	44.2	44.74	-5.19	39.59
**2**	38.1	36.5	1.6	13.6	801	36.5	39.6	41.59	-4.90	36.71
**3**	39.3	38.4	0.9	12.9	762	37.7	41.6	43.37	-4.89	38.51
**4**	40.5	40.4	0.5	14.2	852	39.2	44.3	45.37	-5.32	40.09
**5**	39.5	38.9	0.6	15.1	811	38.3	43.2	43.21	-5.31	37.94
**6**	40.6	38.7	1.9	14.8	874	38.1	42.7	44.68	-5.13	39.59
**7**	39.9	38.7	1.2	14.4	861	38.5	42.8	43.65	-5.10	38.58
**8**	39.8	38.6	1.1	14.4	805	38.1	42.4	42.44	-5.18	37.30
**9**	40.4	39.5	0.9	12.5	829	38.8	43.0	44.27	-5.51	38.81
**10**	39.0	37.7	1.3	12.3	865	37.4	42.1	42.53	-5.20	37.37
**11**	38.0	37.5	0.5	13.9	842	37.4	41.7	42.17	-4.87	37.33
**12**	39.2	38.3	0.8	13.1	776	37.9	42.1	43.65	-5.33	38.36
**13**	39.4	39.1	0.3	14.4	873	38.1	42.3	42.94	-5.30	37.68
**14**	38.6	37.9	0.7	13.5	803	37.7	42.1	42.80	-4.79	38.04
**15**	39.8	37.9	1.9	13.1	898	38.3	42.7	41.90	-5.15	36.79
**16**	41.1	37.8	3.4	14.2	796	38.9	44.1	42.98	-5.17	37.85
**Mean**	39.60	38.46	1.14	13.81	832.6	38.14	42.56	43.27	-5.15	38.16
**±**	0.89	0.92	0.78	0.83	40.18	0.73	1.15	1.08	0.20	1.00
**Min**	38.0	36.5	0.3	12.3	762	36.5	39.6	41.59	-5.51	36.71
**Max**	41.1	40.0	3.4	15.1	898	39.3	44.3	45.37	-4.79	40.09

Abbreviations: D – diopter; Ast. – astigmatism; Ant. – anterior; Elev. – elevation; Post. – posterior; WTW – white-to-white diameter; BFS – best fit sphere; TCP – total corneal power.

A: technical drawing (Above: cross section; below: from above view; measurements in mm); B: photograph of the calotte.; Abbreviations: R – radius; A – axis.

## DISCUSSION

The human cornea provides approximately 75% of the total refractive power of the human eye. There are numerous refractive surgical procedures that target this tissue. Before new techniques can be applied to human eyes, simulations in a comparable setting are desirable. The porcine eye is a commonly used animal model for experimental ophthalmologic surgery, and is very useful in cataract and refractive interventions (2, 9, 10, 19, 20). Only a few studies have offered data on the shape of the porcine cornea (21). However, the histology of the porcine eye is well understood. The porcine cornea is much thicker than that of humans due to a thicker epithelium and stroma (21). In one ex vivo model using laser scanning microscopy, the pachymetry was 1013 ± 10 μm (22). In contrast, in an in vivo model, the pachymetry was only 666 µm (range, 534 to 797). Here, the corneal thickness was measured using ultrasound pachymetry (23). The conflicting results may have been due to the different methods used. The age of the animals may also have influenced these differences, but further study is needed to confirm this possibility. In our study, we found a mean corneal thickness of approximately 833 µm. The range was 762 to 898 µm, which was much higher than reported in the investigation by Faber et al. (23) where a range of 543 to 797 µm was identified. In another study, the mean ex vivo pachymetry was 877.6 µm, which agreed with our results (21).

The corneal diameter varied from 12.3 to 15.1 mm. Our data were very similar to those of Faber et al. (23), who reported corneal diameters between 11.0 and 15.5 mm. Bartholomew et al. (24) reported a vertical diameter of 14.0 mm and a horizontal diameter of 16.6 mm. The higher WTW diameter demonstrated another very meaningful difference between the porcine and the human cornea. Based on an analysis of five porcine eyes, some topographic findings were described before referring to five corneal topographies (21). The mean corneal radius was 8.45 mm, which was close to our findings. However, those authors described higher keratometric results (41.2 D for K1 and 38.8 D for K2) with a wider range (standard deviation, 1.76 to 2.89 D) (21). In this context, our data appeared to be more reproducible. Another experimental study on porcine corneas investigated the influence of the length and depth of astigmatic keratotomies on the corneal topography. Here, single and paired incisions were performed. Using corneal topography, the astigmatic changes were examined to demonstrate the impact of the depth and length of the keratotomies (25). The porcine eye is the most commonly used animal model for ophthalmic surgery. When using the porcine cornea for surgical experiments, researchers must remember that the porcine keratometry is approximately 3 D lower than the human keratometry. The porcine corneal pachymetry of approximately 830 µm is much higher than in humans. An astigmatism of at least 0.5 D can be expected, but values of about 3 D may also be observed. The WTW diameter is commonly over 13 mm. Optical mapping systems like the Orbscan® are suitable devices to measure corneal topography, even in modeling systems such as the porcine eye. 

From our data, we constructed a special calotte, which could be used for ex vivo experimental work with porcine corneal tissue Figure 3. As the collagen fibers are crosslinked using glutaraldehyde, the calotte stabilized the physiological shape of the corneal curvature (13). The calotte is made of polytetrafluorethylene and is very chemical-resistant. The first experimental application confirmed its suitability for experiments requiring a chemical preparation of corneal tissue. Our investigations revealed the typical topography of the porcine cornea. Nevertheless, our study had several limitations. We used an ex vivo model. Although we were careful to adhere to storage methods and limited exposure of the specimens, several factors including post-mortem swelling or mechanisms to reduce swelling may have influenced our measurements. Furthermore, we cannot offer any data concerning the axis of astigmatism. The eyes were examined without consideration for the rotation. By using the Schiotz impression tonometry, we may have influenced the keratometric power. The intraocular pressure has an important impact on the shape of the eye; therefore, a constant and reliable pressure was necessary for the measurements (26). In order to evaluate the therapeutic effect of experimental surgical studies performed on porcine corneal tissue, it is necessary to offer a broad spectrum of basic data. So far, there has been only one study concerning the topographic findings of the porcine cornea. Here, five specimens were studied (21). These reference values should be confirmed by future studies. Our study contributes more data. Nevertheless, further investigation is necessary. Attention to the correct positioning of the porcine eye in the bulb holder and to the constant and reproducible intraocular pressure are necessary to simulate an intraoperative setting. The chosen experimental assembly provided a basis for further investigations, including laser simulations in the porcine cornea. 
